# Learning From eHealth Implementations Through “Implementomics”: A Multidimensional Annotation Model Applied to eHealth Projects of the RAFT Network

**DOI:** 10.3389/fpubh.2019.00188

**Published:** 2019-07-05

**Authors:** Caroline Perrin, Georges Bediang, Mirana Randriambelonoro, Antoine Geissbuhler

**Affiliations:** ^1^HI5lab, Department of Radiology and Medical Informatics, Geneva University, Geneva, Switzerland; ^2^Division of eHealth and Telemedicine, Geneva University Hospitals, Geneva, Switzerland; ^3^Faculty of Medicine and Biomedical Sciences, University of Yaoundé I, Yaoundé, Cameroon

**Keywords:** eHealth, digital health, implementation, annotation model, implementomics, semantics

## Abstract

The implementation of digital health technologies has increased globally, producing substantial amounts of information and knowledge. While there are still areas in digital health that are understudied, concurrently there is an exponential increase in published articles, guidelines, methods, projects, and experiences, many of which fail to reach critical mass (pilotitis). Semantically describing and documenting this implementation knowledge and the effectiveness of these tools will help to avoid the duplication of efforts, to reduce preventable implementation obstacles, and to assure that investments are targeted to the most important technological innovations. The RAFT annotation model, presented in this paper, enables to semantically describe all elements of various outputs and implementation projects that were developed, are used, or are part of the RAFT network. This model was initially developed to annotate various implementations and outputs of the RAFT network to facilitate knowledge documentation and sharing, and to be used as a proof of concept for the *Implementome*. The *Implementome* will be an interconnected knowledge system that enables the user to navigate on multiple dimensions through metadata annotated projects, people, and information, and can serve as base for consensus building, best practices and guidelines. The RAFT annotation model can be further developed to enable the annotation of outputs, implementations, people, initiatives, and projects of the digital health domain in general.

## Introduction

Over the last decade internet, connectivity (1), and the implementation of digital health tools, projects and interventions increased globally, addressing challenges faced by both developed and developing countries in providing accessible, cost-effective, high-quality health care services (2–12). Digital health collectively describes the concepts of eHealth, telemedicine and mHealth, as defined by the World Health Organization (WHO). eHealth is the cost-effective and secure use of information and communication technologies (ICTs) for health and health-related fields, while mHealth is a component of eHealth, and involves the provision of health services and information via mobile technologies (13).

The digital divide between developing and industrialized countries is still prevalent, but connectivity is extending rapidly, including into rural areas (14), facilitating implementations of digital health. In low-resource settings, cellphone-based health education and consultations, personalized health tracking devices, and mobile diagnostic technologies can provide real-time information to improve both individual and public health. Smartphones, e-payment systems, and telemedicine improve access to quality care and more timely deployment of emergency services. ICT innovation, and increased connectivity enables health facilities to enter data directly into central servers through web applications without the need for any software installation or database management at the local level (15).

This deployment of digital health produces voluminous literature on a multiplicity of digital health innovations, and while there are still areas in digital health that are understudied, concurrently there is an increase in published articles, guidelines, methods, projects, and experiences, many of which fail to reach critical mass (pilotitis) (16, 17). The term “pilotitis” is used to express the frustration of many of those in the health sector that the vast majority of digital health projects are limited in scale and undertaken in stand-alone, vertical project mode, with predominantly narrowly focused interventions targeting relatively small populations (18).

Furthermore, terminology is evolving, but definitions are diverging, making it time-consuming and occasionally impossible to find appropriate information. Documenting digital health implementation knowledge and effectiveness will help to avoid duplicating efforts and ensure that investments target meaningful technological innovations. We refer to these developments as “Implementomics,” the ability to capture, organize and exploit the multidimensional knowledge related to implementation issues. As for other “omics” domains, a key challenge is to master the variety and volume of information. Knowledge models can improve this.

The variety and volume of information is challenging in the domain of digital health, but also on a smaller scale as within the Réseau en Afrique Francophone pour la Télémédecine (RAFT) network. Established in 2003, RAFT is a telemedicine and elearning network that is currently operational on four continents. It supports isolated healthcare professionals by providing telemedicine and elearning services using affordable, low-bandwidth technologies. It is not only a platform to share and exchange knowledge, but has implemented a solid local infrastructure to ensure sustainability and maintenance of the network. From experience developed with South-South collaboration to top-down and bottom-up approaches and various certification models, much know-how was produced and various perspectives for improvement were proposed (11, 12, 19, 20).

The model, presented in this paper, enables to semantically describe elements (people, results, etc.) of implementations, articles, courses, tools etc. that were developed, or are part of the RAFT network. It serves as a proof of concept for the *Implementome*, which will be an interconnected knowledge system. The basic building block for the *Implementome* is to associate machine-readable metadata to content.

An example of an annotation model or metadata schema is one for digital photos, which enables to describe, among other properties, the camera used to take the picture, shutter speed, date, and location (21). A useful side-effect of this model is that the same piece of metadata can describe the content, as well as to organize and classify it, therefore setting up other properties that were initially not considered, e.g., the possibility to search for photos of a location, taken at a given time (17).

The example of an annotation model designed for a ward of an oncological hospital illustrates how annotation models can facilitate group decision making in a complex environment, and depicts the crucial role of annotations to address organizational complexity and manage heterogeneous flows of essential data and information (22).

## RAFT Annotation Model

**Figure 1:**
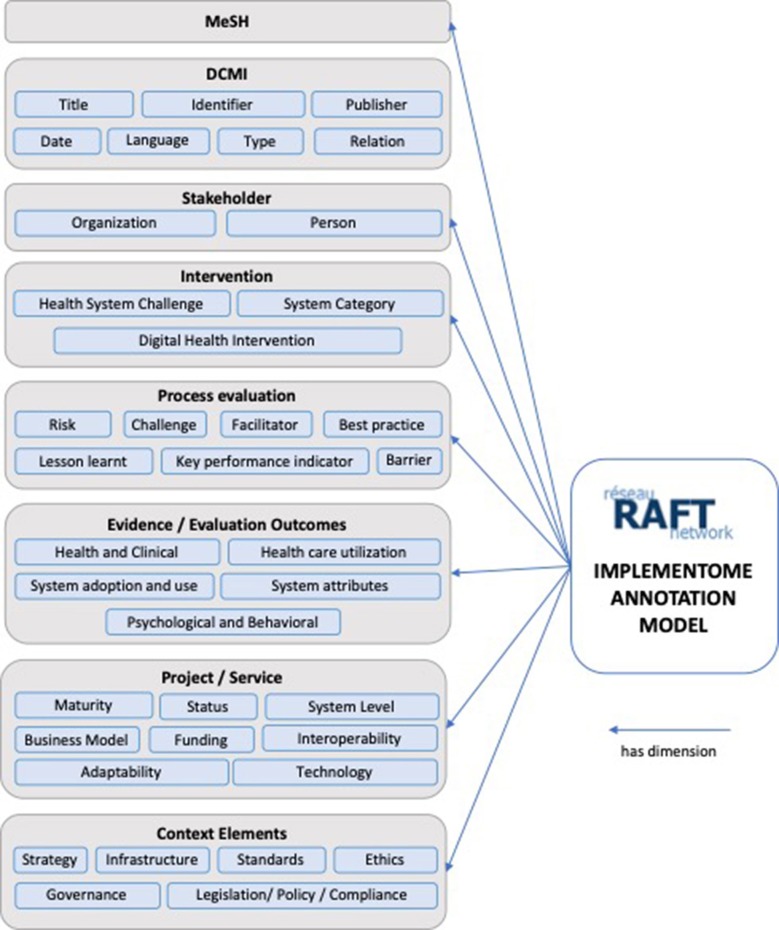
RAFT *Implementome* annotation model.

Annotations are not only a way of explaining and enriching a resource with observations, but are also a means of transmitting and sharing ideas to improve collaborative work practices. The RAFT annotation model was developed to initially enable the annotation of implementations, activities, and outputs of the network. Figure 1 provides an overview on the RAFT annotation model with its eight super-dimensions, and sub-dimensions.

### Description of the Super-Dimensions

#### MeSH (Medical Subjects Headings)

MeSH is a hierarchical controlled vocabulary used for indexing, cataloging, and searching for biomedical and health-related information and documents (23), and in particular the MEDLINE database of biomedical scientific publications. MeSH terminology annotates the entry, ranging from specific diseases, population characteristics, or information science to geographic location.

#### DCMI (Dublin Core Metadata Initiative)

The DCMI was developed to describe web resources and consists of a set of fifteen elements (24). The RAFT annotation model uses seven of these.

- **Title**: given to the resource by the creator or publisher.
- **Date**: indicates a date associated with the creation or availability of the resource.
- **Identifier**: number or string to uniquely identify the resource (e.g., URL or ISBN).
- **Language**: language of the content.
- **Publisher**: entity responsible for publishing.
- **Relation**: reference to a related entry.
- **Type**: the genre or nature of the resource e.g., homepage or journal paper.

#### Stakeholders

This may be either an organization or a person, or both. There may also be several organizations or persons.

- **Person**: affiliated person(s) to a project, or authors of a publication. When available, the Open Researcher and Contributor ID (ORCID) will be included, which aims at uniquely identifying and connecting persons to their contributions across disciplines, borders, and time (25).
- **Organization**: stakeholder organizations in a project, or affiliations of authors for publications.

#### Interventions

Interventions are annotated using the Classification of digital health interventions that was developed by WHO (26).

- **Health System Challenge:** high-level description of needs and addressed challenges in the implementation context.
- **System Category:** describes the types of ICT applications and information systems designed to deliver one or more digital health intervention.
- **Digital health interventions:** organized into four umbrella groupings based on the targeted primary user: (1) Clients; (2) Health care providers; (3) Health system or resource managers; or (4) Data services.

#### Process

The process section annotates implementation aspects. As opposed to outcome evaluations, process evaluation focuses on inputs, activities, and outputs, and evaluating how they work together. Evaluating the process may explain why implementations did or did not work.

- **Challenge**: annotates specific implementation challenges, e.g., resistance to change or change in leadership, but also overarching challenges like political or ethical.
- **Barriers**: annotates barriers like adoption, technical illiteracy, or missing legislation.
- **Risks**: annotates risks like political instability, but also funding continuity.
- **Facilitators**: annotates factors contributing to a successful implementation, e.g., governmental support.
- **Best practices**: annotates a procedure or process that produced optimal results and is established or proposed as a standard suitable for widespread adoption.
- **Lessons learnt**: annotates the learning gained from the process of performing the project or service, e.g., institutional anchoring, or identification of champions.
- **Key performance indicators (KPI):** annotates KPIs, like the number of telemedicine-cases and their status: measuring the number and status of cases is an important KPI and helps identifying if a connected site is having technical or organizational challenges and might need additional support.

#### Evidence/Evaluation Outcomes

Based on a classification from Zanaboni et al. (27), entries are annotated with the following five categories:

- **Health and Clinical**: can be specified with general indicators, disease-specific indicators, or patient-reported outcomes (e.g., improvement in health status, quality of life, medication management, mortality, physical activity, or related to diabetes or hypertension);
- **Psychological and Behavioral:** e.g., patients changing their behavior toward the way they manage their health or a specific disease;
- **Health care utilization:** impact of digital health interventions on the resources involved, including economic effects and time used by patients and providers, and use of the health care system;
- **System adoption and use**: e.g., how patients use a digital health intervention in practice, or the organizational change for health care professionals;
- **System attributes:** other effects focusing on the evaluation of systems themselves, e.g., usability for patients and providers;

#### Project/Service

Can be annotated with

- **Maturity levels:** (*Informal*: early adoption in the absence of formal processes and policies; *Pilot*: Testing and evaluating in a given situation; *Scale-up*: beyond the initial pilot, extension to other populations or centers; *Established operation*: ongoing, since at least 1 year, with funding to continue);
- **Status:** of the project/ service (ongoing, completed and ended, completed and continued, discontinued);
- **System level:** of the implementation (local, regional, national, international);
- **Business model**;
- **Funding:** (Public funding; Donor/non-public funding; Public-private partnership; Private funding).
- **Adaptability**: the project/service may have *broad contextual adaptability*, where it broadly applies to a range of settings and usage scenarios, or it might have *specific adaptability*, where it is only suited to specific needs, users, or geographical localities.
- **Technology**: Hardware, Software (open source, publicly available, proprietary)
- **Interoperability**: according to four interoperability levels defined by the Healthcare Information and Management Systems Society (HIMSS) (28): (1) *Foundational*: basic level of technical interoperability. Data from one IT system can be received by another, but the receiving system does not need to be able to interpret it. (2) *Structural:* intermediate level of technical interoperability, where the data exchanges between IT systems can be interpreted at the data field level, and clinical or operational purpose and meaning of the data is preserved. (3) *Semantic*: highest level of technical interoperability, where two or more systems can exchange information, and the exchanged information can be used. It allows the electronic exchange of patient summary information among caregivers and other authorized parties via potentially disparate electronic health record (EHR) systems. (4) *Organizational*: includes non-technical considerations and enables interoperability that is integrated into end-user processes and workflows in a manner that supports efficiencies, relationships and overall health and wellness through cooperative use of shared data both across and within organizational boundaries.

#### Context Elements

Enables the annotation of context elements that influenced, enabled, challenged, or facilitated the implementation.

- **Strategy**: the entry corresponds, implemented or contributed to a digital health strategy (national or organizational) in the context.
- **Infrastructure**: annotates the need for infrastructure, or leveraging on nationally or private governed infrastructure, or existing services.
- **Legislation/Policy/Compliance**: annotates e.g., compliance with national guidelines or regulatory statuses, or needs for legislation or policy. If there is e.g., no legislation regulating various aspects of telemedicine, questions around medical liability might complexify the implementation significantly.
- **Ethics**: annotates ethical challenges and frameworks.
- **Governance**: annotates national, regional or organizational governance, and Governance challenges, models, and decision-making in acute care.
- **Standards**: annotates need or use of standards.
- **Cybersecurity**: annotates e.g., cybersecurity guidance, mitigation for cybersecurity risks, or to report issues.

**Figure 2:**
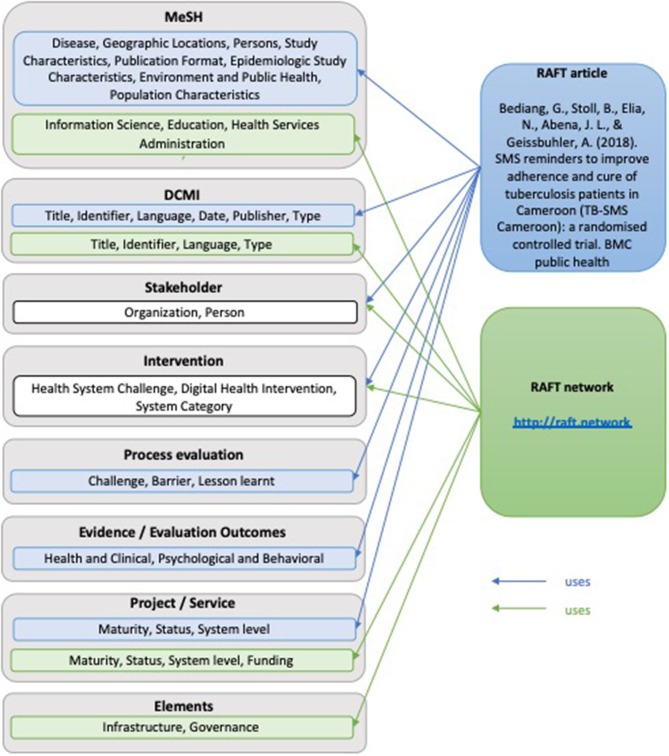
Overview on the application of the annotation model to examples.

For illustrative purposes the annotation model, was applied to two examples (Figure 2). The first one is a journal article reporting on a randomized controlled trial that investigated the effect of SMS reminders on the adherence and cure of tuberculosis patients in Cameroon. The second example is on the RAFT network in general.

### Application of the Model to Examples

#### RAFT Article

- **MeSH Headings**: Tuberculosis, Pulmonary (Diseases); Cameroon (Geographic locations); Adult (Persons); Clinical Trial (Study Characteristics); Randomized Controlled Trial (Epidemiologic study characteristics); Urban Population (Population characteristics); Multicenter Studies as Topic (Environment and Public Health); Journal Article (Publication Format);
- **DCMI**: *Title*: SMS reminders to improve adherence and cure of tuberculosis patients in Cameroon (TB-SMS Cameroon): A randomized controlled trial*; Date*: 2018 May 2; *Identifier*: PMCID: PMC5932834; *Language*: English; *Publisher*: BMC Public Health; *Type*: Journal Article;
- **Stakeholders**: *Person*: Georges Bediang (https://orcid.org/0000-0001-9177-8798); Beat Stoll; Nadia Elia; Jean-Louis Abena; Antoine Geissbuhler (https://orcid.org/0000-0001-5039-3373; *Organization*: Faculty of Medicine and Biomedical Sciences, University of Yaoundé I, Yaoundé, Cameroon; Geneva Tumor Registry, Institute of Global Health, Faculty of Medicine, University of Geneva, Geneva Switzerland; National Tuberculosis Control Program, Ministry of Public Health, Yaoundé, Cameroon; Department of Radiology and Medical Informatics, Faculty of Medicine, University of Geneva, Geneva, Switzerland
- **Intervention:** *Health System Challenge*: 5.3 Low adherence to treatments; *Digital Health Intervention*: 1.1.3 Transmit targeted alerts and reminders to client(s); *System Category*: D Client Communication System;
- **Process Evaluation**: Challenge; Barrier; Lessons learnt;
- **Evidence Evaluation Outcomes**: Health and Clinical, Psychological and Behavioral;
- **Project/Service**: Maturity: informal; Status: completed and ended; System Level: local;

#### RAFT Network

- **MeSH Headings**: Online Social Networking (Information Science); Teaching (Education); Remote Consultation (Health Services Administration)
- **DCMI**: *Title*: Réseau en Afrique Francophone pour la Télémédecine*; Identifier*: http://raft.network; PMCID: PMC5932834; *Language*: English, French, Spanish, Portugese; *Type*: Website
- **Stakeholder:** *Person*: Antoine Geissbuhler (https://orcid.org/0000-0001-5039-3373), Cheick-Oumar Bagayoko; *Organization*: Department of eHealth and Telemedicine, University Hospital of Geneva, Geneva, Switzerland; Department of Radiology and Medical Informatics, Faculty of Medicine, University of Geneva, Geneva, Switzerland; CERTES Expertise Center and telemedicine Research in e-Health, Bamako, Mali;
- **Intervention:** *Health System Challenge*: 2.2 Insufficient supply of services, 2.4 Insufficient supply of qualified health workers, 3.2 Insufficient health worker competence, 3.4 Low health worker motivation, 5.2 Geographic inaccessibility, 6.2 Lack of or inappropriate referrals, 6.4 Delayed provision of care, 6.5 Inadequate access to transportation; *Digital Health Intervention*: 2.4.4 Consultation for case management between healthcare provider(s), 2.6.1 Coordinate emergency response and transport, 2.8.1 Provide training content to healthcare provider(s); *System Category*: S Learning and training system, Y Telemedicine;
- **Project/Service**: Maturity: established operation; Status: completed and ongoing; System level: international, Funding: Public funding, Donor/non-public funding;
- **Elements**: Infrastructure, Governance.

## Discussion

The presented model was developed to annotate activities and outputs of the RAFT network. The purpose of this model is not to replace existing annotations like MeSH, but to connect and extend these to enable a detailed annotation of digital health activities. It will present opportunities for extension when applying it to the digital health domain in general, e.g., annotating health care organizations electronic medical record implementations with the stages of HIMSS Analytics Electronic Medical Record Adoption Model (EMRAM), which measures adoption and utilization of electronic medical record (EMR) functions (29); or the inclusion of technologies like blockchain in the model.

The value of the *Implementome* will be determined by the dependability, and the number and quality of annotated entries. Different strategies exist to populate the *Implementome*. These range from manual annotation by trained experts, crowdsourcing, and hybrid strategies to auto-harvesting of entries.

Crowdsourcing, or “citizen science,” is a strategy for the collection, analysis and sharing of large amounts of data, generally via the Internet. For researchers it represents an opportunity to overcome common barriers to data collection, such as ensuring extensive geographic coverage and maintaining long-term projects. For example, crowdsourcing has been used for decades to harness the power of citizen bird-watchers to better document the distribution and migratory patterns of a wide range of bird species (30). Millions of people around the globe help professional scientists with tasks that range from monitoring changes in local biodiversity to providing innovation and computing power for new drug development.

Manual indexing is precise and trustworthy, but can be time-consuming and costly. Automatic annotation is widely used in genomics (31) and proteomics (32), considering the enormous amount of data shared and classified in these domains. Uniprot, a protein knowledge-based platform, uses two prediction systems the Unified Rule System (UniRule) and the statistical Automatic Annotation System (SAAS) to automatically annotate unreviewed entries in an efficient and scalable manner, in addition to their manual annotation.

MeSH terms in Pubmed are currently manually assigned by human indexers. However, there are consistent efforts in improving automatic indexing (33–35). Probabilistic models and other machine learning algorithms (33, 35) are tested with different sets of data to predict correct MESH terms for documents. MESH Now (34) is an integrated approach using multiple strategies to generate a combined list of candidate MeSH terms for a target article. While automatic classification remains challenging and is undergoing research, the advance in artificial intelligence, specifically in Natural Language Processing and Deep Learning, could lead the way to more precise annotation.

Manual annotation by trained experts produces high quality records but is expensive and time consuming, while entirely automatic strategies are fast and low-cost, but have higher probability for poor quality. As a perspective for the future, the *Implementome* could use a hybrid annotation system with an automatic indexing followed by manual curation.

The main limitation of the proposed RAFT annotation model is that it might not capture all the complexity and detail of the annotated items, as the model uses ontologies to simplify the complicated web of inter-related and resembling terms, and might assign a broader more general term instead of a more detailed concept.

Another limitation comes from the annotations themselves. The model is based on experience of the RAFT network, and is therefore limited to describe well-known processes within the network.

Establishing the possibility to propose additional annotations that will be reviewed and potentially added when implementing the model addresses these limitations. These additional annotations might describe more details, use more accurate terms, or add new emerging concepts.

When implementing the model further limitations or issues might appear like duplicate subjects. To minimize this, the model proposes to use unique identifiers like ORCID ID, or PMCID when possible, however this is not possible for all subjects and needs to be considered in the implementation strategy.

## Conclusion

The presented annotation model enables the collection, annotation and connection of information to encourage the exchange of knowledge and learning, and to facilitate joint knowledge generation to address knowledge gaps, between and across digital health projects, programs and initiatives. This model has been presented and the application was demonstrated. The next step will be to develop the *Implementome*, based on the annotation model and to potentially refine and evolve the model by extending it to the domain of digital health in general.

The vision of the *Implementome* is: (1) to create a central multidimensional digital health implementation hub to facilitate knowledge documentation and sharing, (2) to pool and connect knowledge resources produced by various projects, initiatives and organizations, (3) to enable joint knowledge creation, and (4) to link organizations, academia, people, policymakers, civil society, and other users to digital health knowledge.

To develop the proof-of-concept for the *Implementome* the annotation model will be applied to describe a variety of digital health projects, organizations, tools, and experts, which are identified in a mapping study. These annotated contents will be added to an interconnected knowledge system that will enable the user to navigate on multiple dimensions through metadata annotated contents. The metadata will cover a broad set of elements of relevance for the understanding and processing of the information at different levels of granularity. For example, for an annotated study, it can include information on the regions of the data collection, the study methodology, the intervention, the authors, outcomes, or pointers to related studies.

## Data Availability

No datasets were generated or analyzed for this study.

## Author Contributions

CP, MR, GB, and AG contributed to the conception and development of the annotation model, which is presented and discussed in this paper. CP wrote the first draft of the manuscript and integrated proposed revisions. MR and AG wrote sections of the manuscript. All authors contributed to manuscript revision, read and approved the submitted version.

### Conflict of Interest Statement

The authors declare that the research was conducted in the absence of any commercial or financial relationships that could be construed as a potential conflict of interest.
